# 

**Published:** 1892-06-04

**Authors:** 


### CONTENTS.

Suffering London.—The "Globe's" Parable . . . . . 145

Passing Topics.—Vicarious Benevolence . . . . . 146

The Doctor's Armchair.—Air and Water . . . . . 147

The Attitude of Great Writers towards Disease. III.—French Satirists and the Doctors . . . . . 148

Medical History from the Earliest Times. By E. T. WITHINGTON, M.A., M.B.Oxon. XI.—The Schools of Cos and Cnidus . . . . . 149

The Story of the Insane from Year to Year . . . . 150

Annotations.—Home Life for Working Girls—A Premium on Inefficiency—Mr. Egmont Hake's Book . . . . . 151

The Practitioner's Mirror and Retrospect—

MEDICINE.—The Treatment of Cough in Phthisis . . . 154

SURGERY.—Drainage of the Ventricles in Meningitis . . . 154

THERAPEUTICS.—Bryonia . . . . . 155

OPHTHALMOLOGY.—Sympathetic Ophthalmitis . . . . 156

Notes and News . . . . . 152

Around the Hospitals—Scraps and Gleanings . . . 153

Books Received . . . . . 156

The Institutional Workshop—

HOSPITAL OPINION. — Suffering London. By a HOSPITAL SECRETARY . . . . . 157

HOSPITAL ADMINISTRATION.—Great Ormond Street Children's Hospital . . . . . 157

WITHIN THE HOSPITALS.—Some American Hospitals . . . 158

FURNITURE AND FITTINGS.—Bronchitis or Tracheotomy Kettle —Bucket Fire Extinguisher . . . . . 159

FESTIVAL DINNERS AND HOSPITAL MEETINGS.—City of London Hospital for Diseases of the Chest, 159; North London Hospital for Consumption—National Hospital for the Paralysed and Epileptic . . . . . 160

The Student of Medicine.—Appointments — Vacancies, &c.

### EXTRA SUPPLEMENT.—THE NURSING MIRROR.

En Passant.—Behind Glass Doors—Westminster Hospital Samaritan Fund—Last Offices—American News—Leicester Nursing Institution—National Hospital, Queen Square—Personal Inspection—The Ladies' Sanitary Association—Aberdeen District Nursing Association—Red Blouses and Nurses . . . . lxvii

Ventilation, Disinfection, and Diet. By P. CALDWELL SMITH, M.D. VIII.—Disinfection (*continued*) . . . lxviii

Proposed Bill for the Registration of Midwives . . lxix

Wants and Workers . . . . . lxix

For Reading to the Sick.—The Resurrection . . . . lxix

The Progress of Nursing in Workhouse Infirmaries . lxx

A Letter from Fiji . . . . . lxx

Appointments—Hints to Nurses . . . . . lxxi

The Nurses' Bookshelf.—Obstetric Hints for the Use of Midwives . . . . . lxxi

Everybody's Opinion.—Off Duty—Private Nurses and their Ways—Notes and Queries . . . . lxxi

Off Duty.—Tired of Life (*concluded*) . . . . . lxxii

